# First molecular evidence of *Hepatozoon canis* infection in red foxes and golden jackals from Hungary

**DOI:** 10.1186/1756-3305-7-303

**Published:** 2014-07-02

**Authors:** Róbert Farkas, Norbert Solymosi, Nóra Takács, Ákos Hornyák, Sándor Hornok, Yaarit Nachum-Biala, Gad Baneth

**Affiliations:** 1 Department of Parasitology and Zoology, Faculty of Veterinary Science, Szent István University, Budapest, Hungary; 2 Department of Animal Hygiene, Herd-health and Veterinary Ethology, Faculty of Veterinary Science, Szent István University, Budapest, Hungary; 3 Veterinary Diagnostic Directorate, National Food Chain Safety Office, Budapest, Hungary; 4 School of Veterinary Medicine, Hebrew University of Jerusalem, Jerusalem, Israel

**Keywords:** Fox, Golden jackal, *Hepatozoon canis*, Hungary

## Abstract

**Background:**

Recently, *Hepatozoon canis* infection has been detected among shepherd, hunting and stray dogs in the southern part of Hungary, which is considered to be free of *Rhipicephalus sanguineus* sensu lato and close to the border with Croatia. The aim of this study was to acquire information on the possibility that red foxes and/or golden jackals could play a role in the appearance and spread of *H. canis* in Hungary.

**Methods:**

A conventional PCR was used to amplify a 666 bp long fragment of the *Hepatozoon* 18S rRNA gene from blood samples collected from 334 foxes shot in 231 locations in 16 counties and 15 golden jackals shot in 9 locations in two southwestern counties close to Croatia. A second PCR assay was performed in some of the samples positive by the first PCR to amplify a larger segment (approximately 1500 bp) of the 18S rRNA gene of *Hepatozoon* spp. for further phylogenetic analysis.

**Results:**

*Hepatozoon* infection was detected in canids shot in 30 locations and 9 counties. Altogether 26 foxes (8.0%, 95% CI: 5-11%) and 9 jackals (60%, 95% CI: 33-81%) were PCR positive. *Hepatozoon canis* sequences were obtained from 12 foxes and 7 jackals. DNA sequences from 16 animals were 99-100% similar to *H. canis* from Croatian foxes or dogs while two of the sequences were 99% similar to an Italian fox. Half (13/26) of the infected red foxes and all golden jackals were shot in the two southwestern counties.

**Conclusions:**

This is the first report on molecular evidence of *H. canis* in red foxes (*Vulpes vulpes*) and golden jackals (*Canis aureus*) from Hungary, which is considered free from the tick vector of *H. canis*, *R. sanguineus*. Although no *R. sanguineus* sensu lato had been found on infected or non-infected wild canids, the detection of authochnous canine hepatozoonosis in Hungary might imply that the range of *R. sanguineus* sensu lato has reached this country.

## Background

*Hepatozoon canis* (Eucoccidiorida: Hepatozoidae) is an apicomplexan protozoan species, which is one of the most widespread tick-borne protozoa infecting domestic dogs and wild canids worldwide [1,2]. The life cycle of *H. canis* requires two hosts, merogony occurs in an intermediate vertebrate host, and gametogony and sporogony take place in the haematophagous invertebrate definitive hosts. *Rhipicephalus sanguineus*, commonly referred to as the “kennel tick” or “brown dog tick”, is considered as the major vector of *H. canis*[3]. The role of this tick species, which is the definitive host, as a vector has been reinforced by the evidence of transstadial transmission from tick larvae to nymphs, in addition to transmission from the nymph to the adult stage [4]. Furthermore, transovarial transmission of *H. canis* could not be demonstrated [5].

The occurrence of canine hepatozoonosis is closely related to the geographical distribution of the definitive tick host, which is considered to be one of the most prevalent tick species worldwide [2,6]. In Europe the geographical distribution of *H. canis* is restricted to the Mediterranean region, Balkan, and Iberian peninsulas where *R. sanguineus* sensu lato is frequent [7]. The vector tick becomes infected in the larval or nymph stages by ingesting blood of an intermediate host (dogs and wild canids) containing *H. canis* gamonts within leukocytes. The principal route of infection of dogs or other intermediate hosts is ingestion of a tick or parts of ticks containing mature oocysts, which is different from transmission of other arthropod-borne pathogens transmitted during blood-sucking by vectors (2,3). Salivary transfer of *Hepatozoon* spp. from the final hematophagenous vector host to the vertebrate intermediate host during the blood meal has not been demonstrated [1,2]. The intermediate vertebrate hosts of *H. canis* can also be infected through vertical transmission of the parasite from the bitch to its offspring [8]. Animals from neonatal to adult age can be infected [9]. The infection could be subclinical with low levels of parasitaemia or could be manifested as a severe life-threatening disease with fever, lethargy, anaemia, cachexia, weight loss, and lymphadenopathy with high parasitaemia [10]. Severe co-infections of *H. canis* with other concomitant pathogens transmitted by *R. sanguineus* sensu lato or other vectors are especially frequent involving *Babesia vogeli*, *Ehrlichia canis* and *Anaplasma platys*[9,11].

The potential of *H. canis* to infect a wide range of carnivorous species genetically close to domestic dogs is considerable. Hepatozoonosis has been detected where its tick vector is present in red foxes (*Vulpes vulpes*) in the following European countries: France [12], Portugal [13], Spain [14,15], Croatia [16], and Italy [17]. Infected foxes were also described in Japan [18] and Israel [19]. *Hepatozoon* spp. infection have also been detected in other wild canids such as in the gray fox (*Urocyon cinereoargenteus*) [20], Pampas gray fox (*Lycalopex gymnocercus*) [21], golden jackal (*Canis aureus*) [22,23], African wild dog (*Lycaon pictus*) [24], coyote (*Canis latrans*) [25,26], spotted hyena (*Crocuta crocuta*) [27], cheetah (*Acinonyx jubatus*), leopard (*Panthera iridus*) and lion (*Panthera leo*) [28].

Recently, *H. canis* infection has been detected among shepherd, hunting and stray dogs in the southern part of Hungary close to the border with Croatia, which is considered to be free of *R. sanguineus* sensu lato [29]. The aim of this study was to acquire information on the possibility that red foxes and/or golden jackals could play a role in the appearance and spread of *H. canis* in Hungary.

## Methods

### Collection of samples

**Figure 1:**
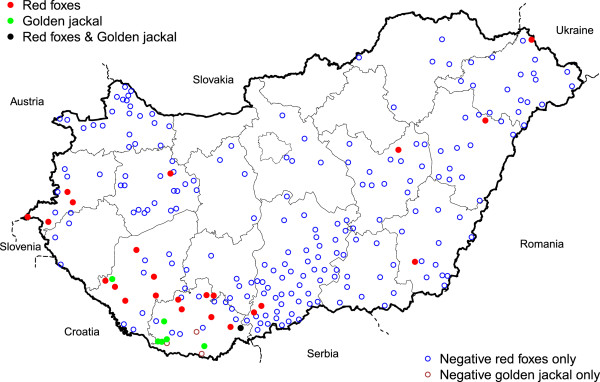
**Geographical locations where red foxes and golden jackals were sampled and *H. canis *** **infected canids occurred in Hungary.**

The blood samples originated from 334 red foxes (*V. vulpes*) representing 231 locations of all seven Hungarian regions (Figure 1). The foxes were shot and the carcasses were sent to the National Food Safety Office, Veterinary Diagnostic Directorate, Budapest as part of a control program on oral immunization of foxes against rabies. Blood samples were also collected from 15 golden jackals (*C. aureus*) shot in nine locations of two southwestern counties close to Croatia belonging to the Southern Transdanubia region (Figure 1). After opening the thoracic cavity of the jackals and foxes, blood was collected from the heart and stored at -18°C. Ticks were searched for only on golden jackals. The study was carried out by observing the hunting regulations of the Hungarian Ministry of Agriculture and Rural Development (No. 79/2004.[V.4.] FVM).

### DNA isolation, amplification and sequencing

DNA was extracted from each blood sample using the QIAamp DNA Mini Kit (QIAgen GmbH., Hilden, Germany) following the “Blood and body fluid” protocol instructions by the manufacturer.

A conventional PCR modified from Inokuma *et al*. [30] was used to amplify a 666 bp long fragment of the *Hepatozoon* 18S rRNA gene with primers HepF (5’-ATA CAT GAG CAA AAT CTC AAC-3’) and HepR (5’-CTT ATT ATT CCA TGC TGC AG-3’). Two and a half μl of extracted DNA were added to 22.5 μl of reaction mixture containing 1.0 U HotStar Taq DNA Plus Polymerase (5 U/μl), 0.5 μl dNTPs (10 mM), 0.2 μl of each primer (50 μM), 2.5 μl of 10× Coral Load PCR buffer (15 mM MgCl_2_ included), 1 μl MgCl_2_ (25 mM) and 17.9 μl DW.

Amplification was performed in a T-personal thermal cycler (Biometra, Goettingen, Germany). An initial denaturation step at 95°C for 5 min was followed by 35 cycles of denaturation at 95°C for 40 s, annealing at 57°C for 40 s and extension at 72°C for 60 s. Final extension was performed at 72°C for 7 min then held at 11°C. DNA of *Hepatozoon* sp. found in a rodent served as positive control. PCR products were visualized under ultra-violet light on 1.5% agarose gel (100 V, 30 min) stained with ethidium–bromide, and were sized by comparison with Gene Ruler 100-bp DNA Ladder (Thermo Fisher Scientific Inc., Waltham, Massachusetts, USA) as molecular marker.

A second PCR assay was performed in some of the samples which were previously positive by the first PCR in order to amplify a larger segment (approximately 1500 bp) of the 18S rRNA gene of *Hepatozoon* spp. for further phylogenetic analysis. This assay used primers HAM-1 F GCCAGTAGTCATATGCTTGTC and HPF-2R GACTTCTCCTTCGTCTAAG [31]. The amplification conditions for this reaction were: 95°C, 5 min; (34× [95°C 20 sec, 56°C, 30 sec, 72°, 90 sec]; 72°C, 5 min).

Selected PCR products were purified and sequenced at Macrogen Inc. (Seoul, South Korea) or at the Hebrew University (Jerusalem, Israel). Sequences were determined in both directions (using the same primers individually as for the PCR). Sequences were compared with 18S rRNA gene sequences of *Hepatozoon* species available in GenBank.

### Phylogenetic analysis

A phylogenetic analysis, which included DNA sequences from the blood of jackals and foxes from the study, was carried out to compare these sequences to *Hepatozoon* spp. sequences that had previously been deposited in GenBank. Sequences were analyzed using the MEGA version 5.1 (http://www.megasoftware.net) and phylogenetic trees were constructed by the Maximum-Likelihood algorithms using the Tamura-3-Parameter model. Bootstrap replicates were performed to estimate the node reliability, and values were obtained from 500 randomly selected samples of the aligned sequence data.

### Statistical analysis

Confidence intervals were calculated by the Sterne method [32]. All statistical analyses and mapping were performed using the R-environment [33].

## Results and discussion

*Hepatozoon* infection was detected in both red foxes and golden jackals shot in 30 locations in 9 counties (Figure 1). Altogether 26 foxes and 9 jackals were PCR positive of which half of the infected red foxes and all golden jackals were shot in two southwestern counties close to Croatia. The other infected red foxes, except for two animals, were shot close to the borders with Austria and Slovenia, Serbia, Romania and Ukraine. The observed prevalence of *H. canis* was 8% (95% CI: 5-11%) in red foxes and 60% (95% CI: 33-81%) in golden jackals. Seven out of 15 golden jackals were infested with *Ixodes ricinus*, *Dermacentor reticulatus* or *Haemaphysalis concinna* ticks.

*Hepatozoon canis* sequences were obtained from 14 red foxes [GenBank: KC886720, KC886722-28, KF322141-144, KJ572978-79], and 9 golden jackals [GenBank: KC886721, KC886729-33, KJ572975-77]. BLAST search revealed that 16 out of 19 sequences obtained in the study were 99-100% similar to *H. canis* from Croatian foxes or dogs while two of sequences [GenBank: KC886722 from a fox and KC886729 from a jackal] were 99% similar to a sequence from an Italian fox [GenBank: GU371447]. A 1545 bp sequence of *H. canis* amplified from a jackal using the HAM-1 F and HPF-2R and comprising almost all of the 18S rRNA gene was submitted to GenBank [KJ634654] and found to be 99% similar to *H. canis* from a Spanish fox [GenBank: AY150067].

**Figure 2:**
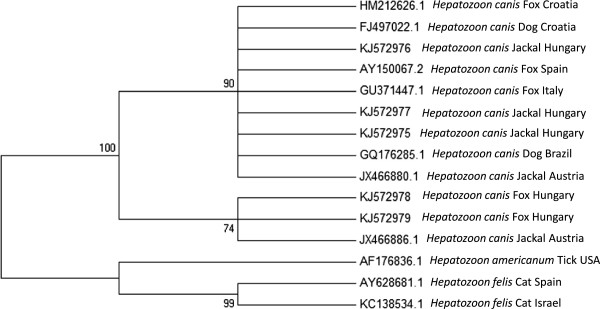
***Maximum likelihood 18S tree; *A *Maximum likelihood *tree phylogram comparing 660 bp *18S Hepatozoon *DNA sequences from Hungarian golden jackals and red foxes to other related *Hepatozoon *spp.** DNA sequences from GenBank. The GenBank accession numbers, *Hepatozoon* species, host species and country of origin from which the sequences were derived are included for each sequence. New sequences derived from the present study are designated as being from Hungary.

A phylogenetic tree constructed by comparison of 660 bp sequences from the 18S rRNA gene (Figure 2) indicated that *H. canis* sequences from the Hungarian jackals and red foxes clustered together with other *H. canis* sequences from dogs, jackals and foxes from other countries in Europe and from Brazil and away from *Hepatozoon americanum* and *Hepatozoon felis* sequences. However, sequences of *H. canis* from Hungarian jackals clustered separately from those of Hungarian foxes, which grouped together with a sequence from an Austrian jackal.

**Figure 3:**
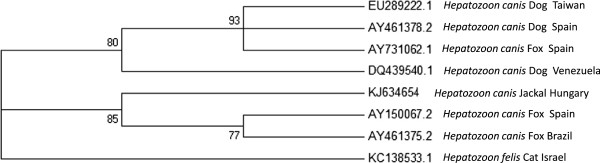
***Maximum likelihood 18S tree; *A *Maximum likelihood *tree phylogram comparing 1382 bp *18S *DNA sequences of *Hepatozoon canis and Hepatozoon felis *to *H. canis *from a Hungarian jackal.** The GenBank accession numbers, *Hepatozoon* species, host species and country of origin from which the sequences were derived are included for each sequence.

A second phylogenetic tree compared 1382 bp sequences of the *Hepatozoon* 18S rRNA gene and included the long sequence of the 18S rRNA gene amplified from a Hungarian jackal [GenBank: KJ634654] (Figure 3). This analysis found that all *H. canis* sequences clustered separately from *H. felis*; however, there were separate clusters within *H. canis*. The *H. canis* sequence from the Hungarian jackal clustered close to *H. canis* from foxes from Brazil and Spain.

*Hepatozoon canis* has been reported previously outside the Mediterranean areas of Europe in imported dogs [34,35]. However, in the last few years, *H. canis* infection of local wild canids was reported for the first time from Slovakia [36], Poland [37] and Austria [23] which were previously considered non-endemic for this parasite due to the absence of *R. sanguineus* sensu lato in these areas. We assume that if these carnivorous species can be sylvatic reservoirs of this apicomplexan parasite they may play a role in the appearance of canine hepatozoonosis in Hungary. Our findings confirm this hypothesis because many red foxes and golden jackals were found to be infected with *H. canis*. Based on the high percentage (60%, 9/15) of the infected golden jackals in the two southern counties neighbouring with Croatia we suppose that they might have brought the parasite to Hungary from that country where dogs and red foxes are infected with *H. canis* and *R. sanguineus* sensu lato is widespread [16,38]. It is unknown how long *H. canis* has been present in Hungary. It is possible that this protozoan parasite arrived in the last decade of the twentieth century when golden jackals started to spread from the Balkan toward Hungary [39], especially from Croatia, and settled in the southwestern part of Hungary [40]. The parasite might have also arrived to the country by infected red foxes from the neighbouring country. In other countries, foxes seem to be very susceptible hosts for this infection, for instance, the prevalence in Portugal was found to be 48% (143/301) in one study [13] and 76% in a second survey [41], in Croatia [16] it was 23% and in Israel, an ELISA survey found that 24% of the foxes were seropositive [19]. However, more data should be collected to answer whether the golden jackal could be more susceptible than the red fox.

Concerning the origin of the parasites detected in Hungary, our hypothesis that this infection came from Croatia was strengthened by the PCR detection of *H. canis* in 13 red foxes and 9 golden jackals shot in the counties neighbouring Croatia. The origin of the local *H. canis* was further substantiated by sequencing *H. canis* of 12 red foxes and 7 golden jackals. Except for three samples, all others were 99-100% similar to *H. canis* from Croatian red foxes or dogs.

The question is where and how these wild animals became infected because *R. sanguineus* sensu lato has not been found in Hungary except for in an imported case [42]. It can be assumed that some golden jackals and/or red foxes might have become infected in Croatia by ingesting *R. sanguineus* ticks containing mature oocysts of *H. canis* or by preying on other animals infested with this vector harbouring infective stages of the parasite. Vertical transmission of the parasite [8] may also help to explain why so many animals were found to be PCR positive. The other question that arose in this study and remains without answer is whether the red foxes shot close to the borders with 5 other neighbouring countries became infected in Hungary or not.

There has been no explanation on how 33 local shepherd, hunting and stray dogs acquired *H. canis*[29]. We think that so many dogs could not be infected by ingesting infected ticks that arrived with golden jackals, red foxes or rodents from the neighbouring countries. Although no *R. sanguineus* sensu lato has been found on infected and non-infected domestic and wild animals, the detection of autochthonous canine hepatozoonosis in Hungary might imply that the range of *R. sanguineus* sensu lato has reached this country. The vector tick might be present at least during the hot summer months suitable for this tick species to establish an endemic focus. In this case the question is whether *R. sanguineus* sensu lato can survive year round and reproduce in Hungary. Further studies should be conducted in the southern part of Hungary in order to answer this question. Another possible explanation is also available. Although the major vector of *H. canis* is *R. sanguineus* sensu lato, the invertebrate host range of this parasite has not yet been fully elucidated. Taking into account that the same tick species can infest dogs [29] and red foxes [43] in Hungary and *H. concinna* nymphs removed from a PCR-negative dog were found positive for *H. canis*[29], the transmission of *H. canis* by other tick species cannot be excluded. In Japan, potential and additional tick vectors, *Haemaphysalis longicornis* and *Haemaphysalis flava* were found [44]. Pfister *et al*. [45] reported that the hedgehog tick, *Ixodes hexagonus* could be a vector of *H. canis*. Forlano *et al*. [46] experimentally proved that *Amblyomma ovale* transmitted the parasite in Brazil. Furthermore, Italian and Austrian scientists suggested the potential role of *I. ricinus* as a vector of *H. canis* among domestic and wild canids [17,23]. However, the results of a later study indicated that *I. ricinus* ingested *H. canis* gamonts during the blood meal but further development and sporogony did not occur suggesting that *I. ricinus* does not act as a vector [47]. *Hepatozoon canis* oocysts have also been found in *Rhipicephalus microplus* (formerly *Boophilus microplus*) but the vector role of this tick species has not been confirmed [48].

Although the first analysis (Figure 2) showed that *H. canis* from Hungarian foxes clustered separately from Hungarian jackals, they did cluster together with *H. canis* from a jackal from neighbouring Austria. Overall, *H. canis* 18S rRNA sequences from different locations and continents including Taiwan in Asia, Brazil in South America, Spain and central European countries, and from different canine hosts such as the domestic dog, golden jackal and red fox, clustered without obvious geographical and host-related division patterns. This may suggest that *H. canis* is a ubiquitous canine parasite which crosses between canine host species and is also transferred successfully between geographic regions by the movement and importation of these hosts.

## Conclusions

To our best knowledge, this is the first report on detection of *H. canis* in red foxes and golden jackals in Hungary. Further investigation should elucidate the routes of *H. canis* transmission amongst wild carnivores and between them and local dogs. Besides *H. canis*, ectoparasite vectors can also transmit several other pathogens from wild canids to dogs. Therefore, the increased activity of vector species due to the climate changes with increasing urbanization and the more common contact between domestic and wild animals stresses the need to study the vector-borne pathogens of red foxes, golden jackals and other carnivore species.

## Competing interests

The authors declare that they have no competing interests.

## Authors’ contributions

Designed the study: RF, SH, GB. Collected samples: RF, SH, ÁH. Processed samples: RF, NT, ÁH. Performed PCR: NT, YNB. Analyzed sequences: RF, GB, NT, YNB. Analyzed the data: NS. Wrote the paper: RF, GB. All authors read and approved the final version of the manuscript.
